# Non-Anticoagulant Heparan Sulfate from the Ascidian *Phallusia nigra* Prevents Colon Carcinoma Metastasis in Mice by Disrupting Platelet-Tumor Cell Interaction

**DOI:** 10.3390/cancers12061353

**Published:** 2020-05-26

**Authors:** Christiane F. S. Silva, Juliana M. Motta, Felipe C. O. B. Teixeira, Angélica M. Gomes, Eduardo Vilanova, Eliene O. Kozlowski, Lubor Borsig, Mauro S. G. Pavão

**Affiliations:** 1 Instituto de Bioquímica Médica Leopoldo de Meis and Hospital Universitário Clementino Fraga Filho, Universidade Federal do Rio de Janeiro, Rio de Janeiro RJ 21941-913, Brazil; chrissobral@gmail.com (C.F.S.S.); jmotta@bioqmed.ufrj.br (J.M.M.); felipecobt@gmail.com (F.C.O.B.T.); epvilanova@gmail.com (E.V.); pavaomsg@gmail.com (E.O.K.); 2 Department of Biomedical Engineering, Lerner Research Institute, Cleveland Clinic, Cleveland, OH 44106, USA; angelica0703@gmail.com; 3 Institute of Physiology and Zurich Center for Integrative Human Physiology, University of Zurich, CH-8057 Zurich, Switzerland; lborsig@access.uzh.ch

**Keywords:** marine invertebrates, glycosaminoglycans, platelets, circulating tumor cells, circulating tumor microemboli, hematogenous metastasis

## Abstract

Although metastasis is the primary cause of death in patients with malignant solid tumors, efficient anti-metastatic therapies are not clinically available currently. Sulfated glycosaminoglycans from marine sources have shown promising pharmacological effects, acting on different steps of the metastatic process. Oversulfated dermatan sulfates from ascidians are effective in preventing metastasis by inhibition of P-selectin, a platelet surface protein involved in the platelet-tumor cell emboli formation. We report in this work that the heparan sulfate isolated from the viscera of the ascidian *Phallusia nigra* drastically attenuates metastases of colon carcinoma cells in mice. Our in vitro and in vivo assessments demonstrate that the *P. nigra* glycan has very low anticoagulant and antithrombotic activities and a reduced hypotension potential, although it efficiently prevented metastasis. Therefore, it may be a promising candidate for the development of a novel anti-metastatic drug.

## 1. Introduction

Metastasis is a multi-step process by which cells from a primary tumor invade the adjacent extracellular matrix, reach the blood or lymphatic vessels, travel through circulation and extravasate the vessel wall to invade a distant tissue and form secondary tumors [1]. During the hematogenous dissemination, tumor cells release cytokines in the bloodstream that activate platelets and the coagulation system [2]. Many glycoproteins at the surface of circulating tumor cells (CTCs) exhibit a specific epitope described as sialyl-Lewis X (or sialyl-Lewis A). These epitopes are recognized and bind to P-selectin expressed on the membrane of activated platelets, which lead to the formation of a platelet cloak around CTCs. Additionally, platelet and tumor cell interaction is reinforced by fibrin accumulation around these cloaks, originating a circulating tumor microemboli (CTM) [3].

CTM protects tumor cells from circulatory mechanical forces and immune cell attack (e.g., natural killer cells) in the bloodstream [4,5]. Both physical stress and immune surveillance make the bloodstream a hostile environment for CTCs. It is estimated that only 0.01% of tumor cells released in the circulation successfully form metastases [6]. Other than mechanically protecting tumor cells, platelets have the intrinsic ability to interact with endothelium, which facilitates the arrest of CTCs in capillaries at metastatic sites, leading to extravasation [7]. In this context, preventing P-selectin binding and the formation of CTMs might decrease the chances of CTC survival in the bloodstream, thus, hindering metastasis formation [8,9].

Heparan sulfate (HS) is a glycosaminoglycan (GAG) expressed in virtually all cells throughout the body. It is mostly found at the cell surface and at the extracellular matrix, forming heparan sulfate proteoglycans (HSPGs) such as Syndecan, Glypican, Perlecan, etc. [10]. Specific interactions of HS with its ligands such as growth factors and receptors, regulate coagulation, inflammation, metastasis, viral infection and many other biological processes [11,12]. Both HS and its analog heparin (HEP) are composed of repetitive disaccharide unities containing α-glucosamine and uronic acid residues linked by (1→4)-glycoside bonds. HS is enriched in *N*-acetyl α-glucosamine→β-glucuronic acid disaccharides bearing different sulfation patterns, whereas HEP is composed mainly of *N*,6-disulfated α-glucosamine→2-sulfated α-iduronic acid disaccharides [13].

GAG-based drugs currently approved for medical use, such as unfractionated (UFH) and low-molecular-weight (LMWH) heparin, chondroitin sulfate (CS) and hyaluronic acid are obtained from tissues of vertebrate animals. Nevertheless, GAG-like polysaccharides found in different marine invertebrates such as clams (mollusks), sea cucumbers (echinoderms) and ascidians (Subphylum: *Urochordata*; Class: *Ascideacea*) have shown promising therapeutic effects in different animal models of human diseases [14,15,16]. In the ascidian *Styela plicata*, for instance, HEP-like glycans were found in intracellular granules of oocyte test cells and hemolymph basophil-like cells [17]. This glycan is enriched in disaccharides containing 2-sulfated α-iduronic acid linked to *N*,6-disulfated α-glucosamine (~75%) or *N*-sulfated α-glucosamine (~25%) and was shown to attenuate inflammation in animal models of colitis. Moreover, this polysaccharide also yielded a nano-derivative able to inhibit proliferation and invasiveness of breast cancer cells [18,19]. Interestingly, the ascidian HEP has significantly lower anticoagulant activity than pharmaceutical UFH from porcine mucosa [20,21].

**Figure 1:**
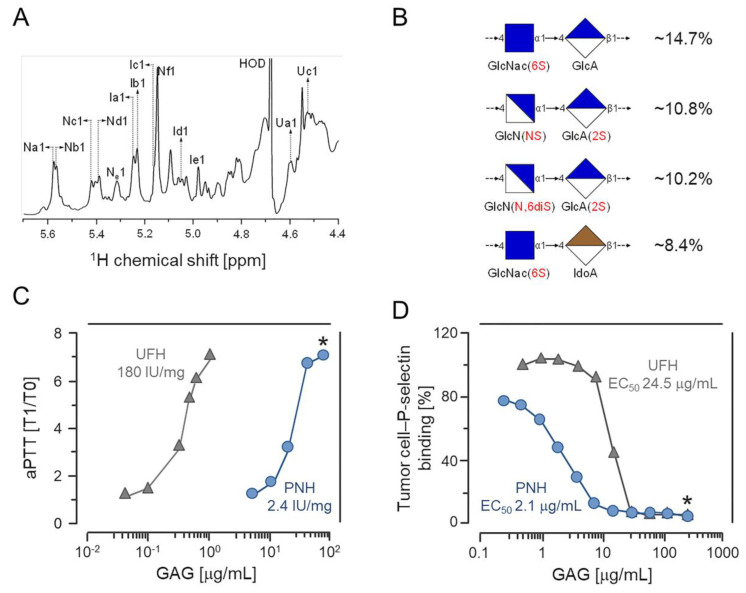
Chemical composition, anticoagulant activity and P-selectin binding blockage by the heparan sulfate from *Phallusia nigra* (PNH). (**A**) ^1^H NMR spectrum of PNH. For details on the signals annotated in the spectra, check reference [21]. (**B**) Proportions of PNH major disaccharides components. (**C**) Anticoagulant activities (expressed as heparin International Units (IU/mg) of PNH (blue circles) and heparin (gray triangles) determined by aPTT clotting assays. (**D**) Doses (EC_50_) of PNH and heparin necessary to inhibit adhesion of LS180 colon carcinoma cells to P-selectin immobilized onto microplate wells. This experiment was repeated and is similar to the one shown in reference [21]. * (*p* < 0.05). Modified (**A**,**C**) or similar (**D**) to reference [21].

Recently, we isolated an HS-like glycan (PNH) from the viscera of the ascidian *Phallusia nigra*. Structural analyses by solution ^1^H and ^13^C nuclear magnetic resonance (NMR) (Figure 1A) showed that PNH is a highly heterogeneous HS enriched in 2-sulfate β-glucuronic and 6-sulfate N-acetyl α-glucosamine units (Figure 1B) [21]. The aPTT assay revealed that PNH has an anticoagulant activity approximately 60-fold lower than UFH (Figure 1C); nevertheless, PNH was still able to inhibit the binding of tumor cells to P-selectin at doses 11-fold lower than UFH, as depicted in Figure 1D [21].

In this study, using mouse models we demonstrate that PNH can prevent lung metastasis of colon carcinoma cells by inhibiting the formation of CTMs. Additionally, we showed that PNH does not activate the coagulation zymogen factor XII (FXII), suggesting its low hypotension potential. Although pharmaceutical UFH and LMWHs have also shown satisfactory P-selectin mediated anti-metastatic activities, they might provoke bleeding in patients [22,23]. Therefore, a compound with ultra-low anticoagulant activity and high efficacy in preventing metastasis, such as PNH, is a promising candidate for therapeutic targeting of P-selectin.

## 2. Results

### 2.1. PNH Has no Cytotoxic Effect on Tumor Cells

**Figure 2:**
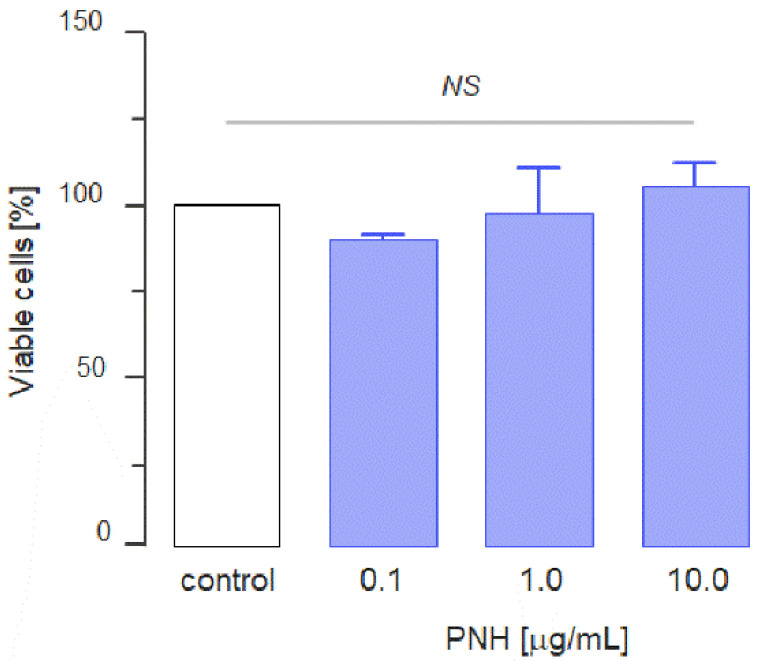
Cytotoxic effect of *P. nigra* heparan sulfate (PNH). 2 × 10^4^ MC-38 cells were cultured in the presence of different concentrations of PNH for 24 h. MTT was added during the last 2 h and the absorbance was measured at 560 nm. The percentage of viable cells was calculated relative to control. Three independent assays were performed and data were compared by analysis of variance (ANOVA); *NS* = no significant statistical difference.

Once we have confirmed the purity and physical-chemical features of the PNH molecule [21], we began to evaluate the in vitro antitumor activity of this molecule. First, we assessed whether PNH has cytotoxic effects on the MC-38 colon carcinoma cell line using an MTT assay. The viability of cells incubated with media supplemented with crescent concentrations of PNH (0.1–10.0 µg/mL) showed no statistically significant differences when compared with those incubated with media without the glycan (control) (Figure 2). This result shows that PNH does not exert in vitro cytotoxic effects on MC-38 cells.

### 2.2. PNH Hinders the Formation of CTMs

**Figure 3:**
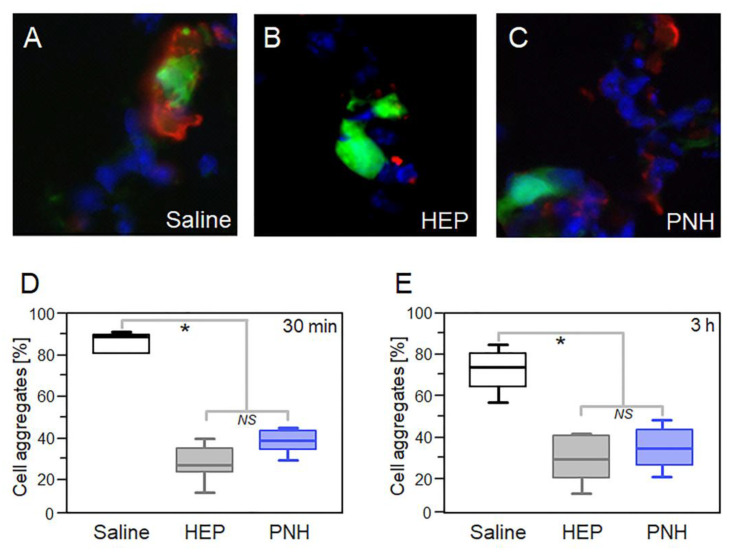
Heparan sulfate from *P. nigra* (PNH) hinders in vivo platelet-tumor cell aggregation. (**A–C**) Aggregates of MC-38GFP colon carcinoma cells (in green [GFP] and blue [DAPI]) and platelets (in red [anti-CD41]) formed in the lung microvasculature of mice were analyzed by immunofluorescence. Quantification of aggregates present in the lungs of animals treated during 30 min (**D**) or 3 h (**E**) with saline (white squares), 1 mg/kg PNH (blue) or 20 mg/kg porcine heparin (gray). Results were expressed as percentages of aggregated tumor cells (20 fields per lung, three animals per treatment) and compared by analysis of variance (ANOVA); NS (no statistical significance) and * (*p* < 0.05).

The interaction of circulating tumor cells with platelets is responsible for the formation of CTMs and is essential to the successful seeding at metastatic sites [3]. This interaction is primarily mediated by P-selectin and our results showed that PNH strongly inhibits the binding of tumor cells to immobilized P-selectin in vitro (Figure 1D). Hence, the ability of PNH to prevent the formation of CTMs was assessed by quantifying the aggregation of GFP-positive MC-38 cells (MC-38GFP) to activated platelets in the lung microvasculature (Figure 3A–C). C57BL/6 mice were intravenously injected (i.v. injection) with PNH (1 mg/Kg) or UFH (20 mg/kg) 10 min before i.v. injection of MC-38GFP cells. After 30 min (Figure 3D) or 3 h (Figure 3E), tumor cells-platelets complex was quantified in lung sections by immunofluorescence. We used a dose 20 times lower of PNH on this experiment, because our in vitro analyses showed that PNH inhibits adhesion of LS180 colon carcinoma cells to P-selectin more efficiently than HEP. Figure 3 shows that at both time points, the lung capillaries of the animals treated with the GAGs presented fewer aggregates than those treated with saline. Furthermore, platelets-tumor cell aggregation was inhibited to the same extent by PNH and HEP, despite the use of a much lower dose of PNH.

### 2.3. PNH Prevents Lung Metastases of Colon Carcinoma Cells in Mice

**Figure 4:**
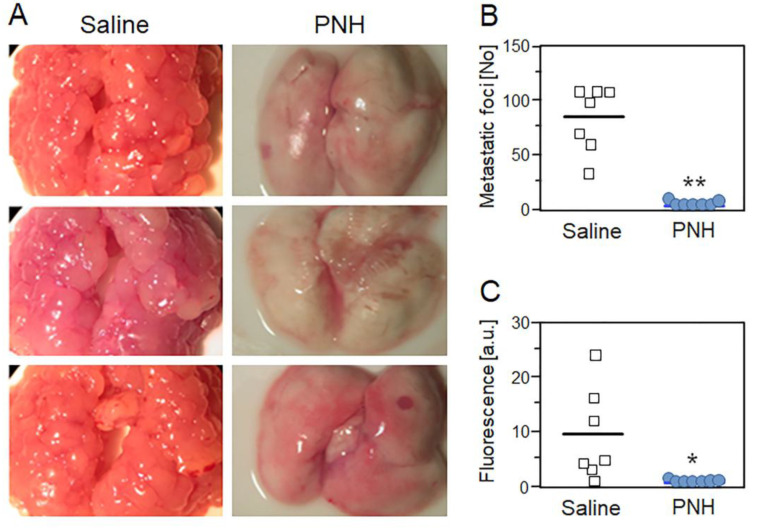
*P. nigra* heparan sulfate (PNH) prevents lung metastasis of colon carcinoma in mice. Representative images (**A**), metastatic foci (**B**) and fluorescence quantification (**C**) of lung metastases present in mice treated with a single dose of PNH (1 mg/kg; blue circles) or saline (white squares) 15 min before injection of MC-38GFP colon carcinoma cells. Results (seven animals per group) were compared by *t*-test; * (*p* < 0.05) and ** (*p* < 0.001).

Because metastasis efficiency depends on the platelet and tumor cell association, we evaluated the efficacy of PNH in preventing the onset of lung metastasis in mice 28 days after i.v. injection of GFP-positive MC-38 cells (Figure 4A). Both macroscopic counting of metastatic foci (Figure 4B) and fluorescence quantification present in lung homogenates (Figure 4C) showed that a single dose of PNH (1 mg/kg), administered intravenously 15 min before the injection of MC-38 cells, dramatically reduces (*p* < 0.05) the incidence of lung metastases relative to control (up to 100 foci per lung observed in animals treated with saline) (Figure 4A–C). Considering that it has no cytotoxic effect on MC-38 cells (Figure 2), the high anti-metastatic activity may be directly related to the ability of PNH to prevent P-selectin mediated formation of CTMs, decreasing their survival in the bloodstream and preventing metastatic colonization.

### 2.4. PNH Has Low Anticoagulant and Antithrombotic Activities and Hypotension Potential

**Figure 5:**
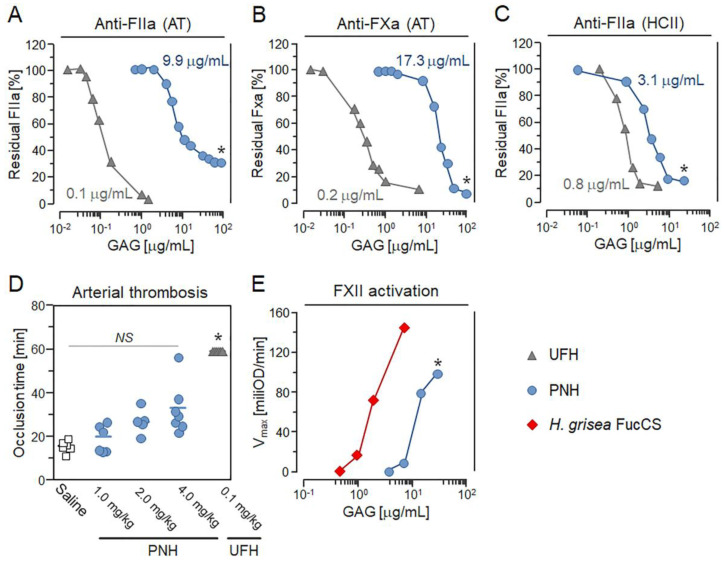
Anticoagulant, antithrombotic and pro-FXIIa activities of the heparan sulfate from *P. nigra* (PNH). Doses (EC_50_) of PNH and UFH promoting in vitro AT-mediated anti-FIIa and anti-FXa (**A**,**B**) and HCII-mediated anti-FIIa (**C**) activities. Results expressed as IU/mg and EC_50_ in panels A–C (means, three independent assays) were compared by *t*-test. Gray triangles—UFH and blue circles—PNH. (**D**) Effects of saline (white squares), UFH (0.1 mg/kg) and crescent doses of PNH (1→4 mg/kg) on the thrombus formation in arteries of mice. Occlusion times (seven animals per condition) were compared by analysis of variance (ANOVA). (**E**) FXII activation promoted by PNH (blue circles) and *H. grisea* FucCS (red diamonds) was estimated by assessing in vitro activation of prekallicrein present in human plasma with basis on the increase in absorbance (405 nm) promoted by the chromogenic substrate for kallikrein; results expressed as optical density/min (mean, three independent assays) were compared by *t*-test. *NS* (no statistical significance) and * (*p* < 0.05).

The major challenge of using HEPs as antitumoral or anti-metastatic agents is the large risk for bleeding and hemorrhage due to their strong anticoagulant activity. For this reason, we evaluated the potential of PNH to cause hemorrhage by assessing its anticoagulant and antithrombotic activity. Our previous study showed that PNH slightly increased the clotting time, as shown in (Figure 1C) [21], and its anticoagulant activity was determined as 2.46 IU/mg, around 60-fold lower than that of UFH (~180 IU/mg). Therefore, we evaluated the ability of PNH to potentiate antithrombin (AT) or heparin cofactor II (HCII)-mediated factor IIa or factor Xa inhibition. The EC_50_ of PNH for AT-mediated FIIa and FXa inhibition was 9.98 and 17.32 µg/mL, respectively, far higher than UFH (EC_50_ = 0.10 and 0.22 µg/mL) (Figure 5A,B). Likewise, the EC_50_ of PNH for HCII-mediated FIIa inhibition was 3.18 µg/mL, four-fold higher than that of UFH (EC_50_ = 0.79 µg/mL) (Figure 5C).

Next, we determined whether PNH has antithrombotic activity in vivo. We employed a mouse model of ferric chloride-induced arterial thrombosis, and evaluated carotid artery occlusion time after injury [20]. Animals treated intravenously with 1 or 2 mg/kg of PNH showed similar occlusion time when compared to those receiving saline (control) (Figure 5D). Nevertheless, a higher dose of PNH (4 mg/kg) was able to double the occlusion time compared to the control, but it was significantly less effective (*p* < 0.05) than UFH administered at 40-fold lower doses (0.1 mg/kg) (Figure 5D). These results indicate that the metastasis-preventing dose of PNH (1 mg/kg) has a minimal antithrombotic effect in mice.

Previous reports have shown that the fucosylated chondroitin sulfate (FucCS), a GAG composed of a CS core decorated with branches of sulfated fucose, purified from the body-wall of the sea cucumber *Holothuria grisea* may provoke kallikrein-mediated hypotension by activating FXII (FXIIa) [15]. Hence, we used a chromogenic assay to evaluate the potential of PNH to cause FXIIa-triggered hypotension by comparing its ability to promote the generation of kallikrein relative to the *H. grisea* FucCS. Our in vitro chromogenic assays demonstrate that PNH is approximately six-fold less potent (*p* < 0.05) than *H. grisea* FucCS in activating prekallikrein (Figure 5E).

## 3. Discussion

Although metastasis is the main cause of mortality for most malignant tumors, therapeutic agents targeting its prevention are not clinically available yet. Current treatment of metastatic tumors is mostly based on surgical excision and/or chemotherapy, which often present poor outcomes in prolonging patient survival [24,25]. Nevertheless, the search for new therapeutic compounds aiming different events of the metastatic cascade has shown promising results [25]. PNH from the ascidian *P. nigra* demonstrated, in this work, a significant anti-metastatic activity associated with low bleeding and hypotensive potential, suggesting that it is a strong candidate for development of a novel therapeutic agent for metastatic tumor treatment.

HSs from different sources should be studied as a family of related polysaccharides rather than a single GAG type considering their significant compositional variability [26]. Despite the intrinsic heterogeneity, the distinct disaccharide components of mammalian HSs are often arranged as repetitive building-blocks along their chains [26]. Previous two-dimensional (2D) NMR spectra assessments performed by our group have already demonstrated that PNH possesses increased proportions of disaccharides containing *N*-acetyl α-glucosamine and/or β-glucuronic acid (>50%), which is characteristic of HSs, but they are randomly arranged along their chains [21].

The primary GAG mechanism for metastasis prevention relies on disrupting the P-selectin-mediated interaction between CTCs and platelets, compromising the formation of CTMs, which are responsible for improving tumor cell survival during hematogenous dissemination [27]. DSs from *S. plicata* and *P. nigra*, which are mostly composed of disulfated disaccharides containing 2-sulfated α-iduronic acid linked to 4-sulfated or 6-sulfated *N*-acetyl α-galactosamine, can inhibit P-selectin at similar doses (IC_50_ = 13 µg/mL), whereas porcine DS, enriched in monosulfated disaccharides of α-iduronic acid→*N*-acetyl α-galactosamine 4-sulfated (up to 95%), is ineffective [8]. Likewise, HS purified from the viscera of the scallop *N. nodosus,* which presents increased proportions of monosulfated β-glucuronic acid→*N*-acetyl α-glucosamine disaccharides (>70%), also inhibits P-selectin (IC_50_ ~ 30 µg/mL) [28]. Both PNH and other GAGs with low sulfate content have already shown higher efficacy to inhibit P-selectin compared to the highly anionic UFH [22,29]. For this reason, the capacity of distinct GAGs to bind to P-selectin seem to be related to the presence of oligosaccharide sequences bearing specific conformational features (e.g., ring shapes, torsion angles and spatial distribution of sulfate groups) along their chains rather than relying exclusively on anionic strength [30]. Furthermore, in the present study, we demonstrated that PNH has also higher efficacy in preventing the formation of CTMs than UFH (20-fold), DSs from *S. plicata* and *P. nigra* (four-fold) and *N. nodosus* HS (eight-fold), indicating stronger anti-metastatic potential than other GAGs obtained from vertebrates or invertebrates [22,28,29].

As observed for PNH during our in vivo experiments, DSs from *S. plicata* and *P. nigra*, as well as FucCS from the sea cucumber *H. grisea,* also attenuate (>95%) lung metastases of MC-38 colon carcinoma cells in mice [8,31]. Similarly, *N. nodosus* HS is effective in decreasing (>75%) the incidence of lung metastases of LLC lung carcinoma cells injected intravenously in mice [28]. Despite being less effective, UFH and LMWHs also exert satisfactory P-selectin-mediated anti-metastatic activities [22,23]. Furthermore, a few clinical trials indicate that administration of UFH and LMWHs dalteparin, nadroparin and certoparin might bring benefit clinical outcomes to patients with different solid tumors in advanced stages [32]. Although we did not evaluate whether PNH can modulate other pathways in cancer, where GAG treatment has shown positive outcomes, such as E-cadherin up-regulation and HGF, heparanase and galectin-3 inhibition, its structural resemblance with other HSs/HEPs allows us to speculate that PNH could exert pharmacological effects in different events of the metastatic cascade and might act as anti-metastatic agent in a synergistic manner [22,33,34,35,36].

The anticoagulant activity of UFH is due to the AT/HCII-mediated inactivation of several coagulation system enzymes, especially FIIa, and FXa [37]. Our in vitro assays revealed that PNH exerts a negligible anticoagulant activity, mostly mediated by HCII. This reduced AT-mediated anticoagulant potency may occur due to the absence of the specific pentasaccharide sequence present in UFH, which promotes the conformational change responsible for potentiating AT [37,38]. Despite presenting mostly a serpin-independent anticoagulant activity, the FucCS from *H. grisea* also activates HCII; nevertheless, its activity (~50 IU/mg) is far higher than that obtained with PNH [15]. *N. nodosus* HS is also more anticoagulant (38 IU/mg) than PNH [28]. On the other hand, DSs from *S. plicata* and *P. nigra* have anticoagulant activities (~8.0 and ~0.5 IU/mg, respectively) similar to PNH [8]. Some low-molecular-weight HEP derivatives with very-low-anticoagulant activities (up to 1 IU/mg anti-Xa activity) were also able to attenuate metastasis in mice, but at higher doses (up to 20-fold) than PNH [39].

Pharmaceutical doses of UFH and LMWHs required to effectively inhibit either P-selectin or other therapeutic targets of the metastatic cascade may provoke bleeding on patients [22,23]. Previous attempts to develop HEP derivatives, as well as semi-synthetic GAG-like oligosaccharides (SAGEs), which are depleted of anticoagulant activity but effective in preventing metastasis, have shown poor pharmaceutical outcomes [22,23,40]. Therefore, the low-anticoagulant GAGs with high anti-metastatic activities found in marine invertebrates, especially PNH and DSs from ascidians, are attractive sources of novel drugs to prevent metastasis. Moreover, the low but detectable antithrombotic effect of PNH might also attenuate the procoagulant state commonly observed in cancer patients without increasing the bleeding risk [32]. In addition, the low ability to activate kallikrein, involved in hypotension events, is an advantage of PNH compared to other GAGs [15].

Notwithstanding the pharmacological potential, the production of GAG-based drugs such as PNH on an industrial scale relies on a constant supply of large amounts of animal raw material. The ascidian *Ciona intestinalis*, which has been mass-produced via aquaculture as a source of biomass to produce biogas by Swedish and Norwegian companies, presents in their viscera anti-metastatic DS similar to that found in *P. nigra* [41]. Moreover, scallops of the genus *Nodipecten* containing HS effective in preventing metastasis are largely cultivated for food purposes in marine farms spread around the world [28,42]. Considering that the viscera of both *C. intestinalis* and *N. nodosus* are underused or discarded, their use would not affect the target products of the aquaculture industry, and thus, the current farming should be fully capable of supplying marine raw material for manufacturing novel GAG-based anti-metastatic drugs.

## 4. Material and Methods

### 4.1. Samples and Cell Lines

DS and HEP/UFH from porcine mucosa and 4-sulfated (CS-4S) and 6-sulfated (CS-6S) CSs from porcine or shark cartilage, employed as GAG’s standards, were purchased from Sigma-Aldrich (St. Louis, MO, USA). The 6th international Heparin Standard (2154 UIs per vial, Lot No. 07/328) was obtained from the National Institute for Biological Standards and Control (Potters Bar, UK). The FucCS from *Holothuria grisea* was kindly provided by Dr. Gustavo Santos (Federal University of Rio de Janeiro, Rio de Janeiro, Brazil). Human colon carcinoma cells (LS180) purchased from ATCC (Manassas, VA, USA) were grown in minimum essential medium-α (Invitrogen; Carlsbad, CA, USA) supplemented with 10% FBS (Invitrogen). Mouse colon carcinoma cells (MC-38) expressing green fluorescent protein (MC-38GFP) [8], provided by Dr. Lubor Borsig, were grown in Dulbecco’s modified Eagle’s medium with 4.5 g/L glucose (Sigma-Aldrich) supplemented with 10% FBS (Invitrogen). All reagents were purchased from Sigma-Aldrich unless otherwise stated.

### 4.2. Isolation and Purification of PNH

Adult specimens of the ascidian *P. nigra* were collected in the Ilha Grande Bay (Rio de Janeiro state, Brazil) by scuba diving. Sulfated polysaccharides from the viscera of *P. nigra* were extracted through proteolytic digestion with papain, and then, PNH was purified with anion-exchange chromatography, as previously described [21,43]. Briefly, crude polysaccharide extracts from *P. nigra* were applied into a DEAE-cellulose column, equilibrated with 50 mM sodium acetate (pH 5.0), and then eluted through a linear gradient of 0.5→1.0 M NaCl. Fractions of 4 mL were collected and checked for metachromasy [43]; four peaks were identified at different NaCl concentrations. Fractions containing each peak were pooled together, dialyzed against distilled water, lyophilized and stored at −20 °C for further utilization. PNH disaccharide composition was previously shown by NMR analysis [21].

### 4.3. Cell Viability Assay

To evaluate cytotoxicity of PNH, an MTT reduction assay was performed. 2 × 10^4^ MC-38 cells were seeded to a 96-well plate (Jet Biofil, Guangzhou, China) and cultured with 0.1; 1.0 or 10.0 µg/mL of PNH for 24 h (200 µL of final volume). Then, 500 µg of MTT was added to the wells and incubated for 2 h at 37 °C in an atmosphere of 5% CO_2_. After this period, MTT was solubilized with DMSO and the absorbance was read in a microplate reader (560 nm). Cells cultured without PNH were identified as the control group and its absorbance was considered 100% of viability. This experiment was done in triplicate.

### 4.4. In Vivo Platelet-Tumor Cell Aggregation

Assessments of in vivo formation of tumor cell-platelet aggregates were performed as previously described [8]. C57BL/6 mice of 8–12 weeks old (~25 g; both sexes) were anesthetized via intramuscular injection of 10 mg/kg ketamine (Cristalia; São Paulo, Brazil) and 1.6 mg/kg xylazine (Bayer; São Paulo, Brazil). Subsequently, PNH (1 mg/kg), UFH (20 mg/kg) or saline (control) were intravenously administered in their tail vein, followed by a tail vein injection of 3 × 10^5^ MC-38GFP cells 10 minutes later. Lungs from animals euthanized 30 min or 3 h after treatment were analyzed by immunofluorescence using an Axio Imager A1 microscope (Zeiss; Oberkochen, Germany). Frozen sections of the lungs were incubated with rat anti-mouse CD41 antibody (BD Life Sciences; Franklin Lakes, NJ, USA) and then by goat anti-rat Alexa 568-conjugated antibody for labeling the platelets. Twenty fields/lung section in four sections per lung were analyzed. The number of aggregates present in animals treated with PNH, UFH, and saline was compared by ANOVA followed by Bonferroni post-test using Origin 8.0 software. All the in vivo assays were performed by following, in a strict manner, the guidelines of our institution (Federal University of Rio de Janeiro) for animal care and experimentation approved by the local ethical committee (approval number 01200.001568/2013-87, protocol 054/19).

### 4.5. Experimental Metastasis Model

The anti-metastatic effect was evaluated by quantifying lung metastases in C57BL/6 mice treated intravenously with a single dose of PNH (1 mg/kg) or saline (control) 15 min before injection of 3 × 10^5^ MC-38GFP cells. After 28 days, animals were euthanized and macroscopical metastatic foci present in their lungs were counted. Measurements of fluorescence emitted by MC-38GFP cells present in lung homogenates were performed as described elsewhere [8]. The anti-metastatic efficacy of PNH compared to the control group was analyzed by *t*-test using Origin 8.0 software (OriginLab; Northampton, MA, USA).

### 4.6. In Vitro Anti-FIIa and -FXa Activities

PNH and UFH were subjected to FIIa and FXa amidolytic activity assessments by measuring the hydrolysis of chromogenic substrates [32]. AT (50 nM) or HCII (68 nM) from Hematologic Technologies (Essex Junction, Chittenden, VT, USA) were incubated in TS/PEG buffer (0.02 M Tris/HCl, 0.15 M NaCl and 1.0 mg/mL polyethylene glycol 8000, pH 7.4) and then 2 nM FIIa or FXa (Hematologic Technologies) was added to trigger the reaction. After incubation (60 s at 37 °C), residual FIIa or FXa activities were determined by adding 100 μM of chromogenic substrates S-2238 or S-2765, respectively (Chromogenix; Molndal, Sweden), and then recording absorbance (405 nm) during 300 s in a ThermoMax Microplate Reader (American Devices; Sunnyvale, CA, USA). Anti-FIIa and -FXa potencies of PNH and UFH (IC_50_) were compared by *t*-test using Origin 8.0 software (OriginLab).

### 4.7. In Vivo Antithrombotic Activity

The effectiveness of PNH in preventing arterial thrombosis was evaluated with an in vivo model [20]. Carotid arteries of C57Bl/6 mice isolated by surgical dissection were placed under an ultrasonic probe (Transonic System; Ithaca, NY, USA) for monitoring blood flow and then PNH (1→4 mg/kg), UFH (0.1 mg/kg) and saline (control) were administered intravenously to the animals. Thrombus was induced by laying a filter piece soaked with 10% ferric chloride over the isolated artery for 3 min and then monitored for 60 min or up to the complete occlusion of the artery (occlusion time). Antithrombotic activities of PNH and UFH were compared by ANOVA using Origin 8.0 software (OriginLab).

### 4.8. FXII Activation Assay

Different concentrations of PNH or FucCS from *H. grisea* were incubated with 40 μL human plasma diluted in TS/PEG (three times). After incubation (60 s at 37 °C), activation of FXII was indirectly assessed by measuring the conversion of plasma prekallikrein with 0.3 mM kallikrein chromogenic substrate S-2302 (Chromogenix) and then recording the absorbance (405 nm) for 300 s. The efficacy of the GAGs in activating FXII was calculated based on the rate of p-nitroanilide formation [15].

## 5. Conclusions

Compounds such as the S-nitrocaptropil and some PEGylateted-thioaptamers (ESTAs) were shown to impair metastasis by inhibiting P- or E-selectin in preclinical evaluations [44,45]; nevertheless, none of them has shown satisfactory progress in clinical trials thus far. The comprehensive set of in vitro and in vivo assays presented here shows that PNH purified from the viscera of the ascidian *P. nigra* has high anti-metastatic activity and low bleeding and hypotensive potential; therefore, it could be an interesting candidate to be tested in future clinical trials.
